# Ecological momentary assessment of using food to soothe during infancy in the INSIGHT trial

**DOI:** 10.1186/s12966-019-0837-y

**Published:** 2019-09-05

**Authors:** Elizabeth L. Adams, Michele E. Marini, Timothy R. Brick, Ian M. Paul, Leann L. Birch, Jennifer S. Savage

**Affiliations:** 1 Center for Childhood Obesity Research, 129 Noll Laboratory, The Pennsylvania State University, University Park, PA 16802 USA; 2 Department of Nutritional Sciences, Penn State University, University Park, PA USA; 3 Department of Human Development and Family Studies, Penn State University, University Park, PA USA; 4 Department of Foods and Nutrition, University of Georgia, Athens, GA USA; 5 Pediatrics and Public Health Sciences, Penn State College of Medicine, Hershey, PA USA

**Keywords:** Obesity prevention, Responsive parenting, Infant cry, Infant fuss, Infant feed, Soothing strategies

## Abstract

**Background:**

Use of food to soothe infant distress has been linked to greater weight in observational studies. We used ecological momentary assessment to capture detailed patterns of food to soothe and evaluate if a responsive parenting intervention reduced parents’ use of food to soothe.

**Methods:**

Primiparous mother-newborn dyads were randomized to a responsive parenting intervention designed for obesity prevention or a safety control group. Responsive parenting curriculum included guidance on using alternative soothing strategies (e.g., swaddling), rather than feeding, as the first response to infant fussiness. After the initial intervention visit 3 weeks after delivery, mothers (*n* = 157) were surveyed for two 5–8 day bursts at infant ages 3 and 8 weeks. Surveys were sent via text message every 4 h between 10:00 AM-10:00 PM, with 2 surveys sent at 8:00 AM asking about nighttime hours. Infant fusses and feeds were reported for each 4-h interval. Food to soothe was defined as “Fed First” and “Not Fed First” in response to a fussy event. Use of food to soothe was modeled using random-intercept logistic regression.

**Results:**

The control group had greater odds of having Fed First, compared to the responsive parenting group at ages 3 and 8 weeks (3 weeks: OR = 1.9; 95% CI = 1.4–2.7; *p* < 0.01; 8 weeks: OR = 1.4; 95% CI = 1.0–2.1; *p* = 0.053). More responsive parenting mothers reported using a responsive parenting intervention strategy first, before feeding, than controls at ages 3 and 8 weeks (3 weeks: 58.1% vs. 41.9%; 8 weeks: 57.1% vs. 42.9%, respectively; *p* < 0.01 for both). At both ages combined, fewer fusses from responsive parenting infants were soothed best by feeding compared to controls (49.5% vs. 61.0%, respectively; *p* < 0.01). For both study groups combined, parents had greater odds of having Fed First during the nighttime compared to the daytime at both ages (3 weeks: OR = 1.6, 95% CI = 1.4–1.8; *p* < 0.01; 8 weeks: OR = 2.1; 95% CI = 1.7–2.6; *p* < 0.01).

**Conclusions:**

INSIGHT’s responsive parenting intervention reduced use of food to soothe and increased use of alternative soothing strategies in response to infant fussiness. Education on responsive parenting behaviors around fussing and feeding during early infancy has the potential to improve later self-regulation and weight gain trajectory.

**Trial registration:**

NCT01167270. Registered July 21, 2010.

## Introduction

During infancy, parental feeding practices shape infant eating behaviors by determining what, when, how, and how much infants are fed [1]. During the first 3 months after birth, crying is an infant’s primary form of communication, reaching maximum intensity around age 8 weeks [2]. This fussiness may be perceived as a cue that infants are hungry [3, 4]; however, infants cry for many reasons other than hunger (e.g. tired, wet, overstimulated). The use of “food to soothe” to regulate emotions or calm includes feeding in response to infant distress or fussiness that is unrelated to hunger [5, 6]. Infants who are perceived as more fussy or negative may be exposed to greater use of food to soothe [5, 7, 8]. Using food to soothe has been associated with negative health outcomes such as rapid weight gain and greater weight status during infancy [5, 6, 9], as well as increased emotional eating [10], less healthy dietary patterns (e.g. energy dense snack foods, less fruits and vegetable intake) [11, 12], and eating in the absence of hunger during later childhood [13].

In contrast, parental use of responsive feeding supports the development of infant appetite regulation [14]. Responsive feeding includes prompt, contingent, and developmentally appropriate responses to infant cues of hunger and fullness [15]. This includes feeding when infants are showing signs of hunger and stopping feeding when infants are showing signs of fullness. When infants are fussy, but not showing signs of hunger, responsive parenting includes the use of alternative soothing strategies such as rocking, swaddling, and white noise, instead of feeding. Responding to non-hunger related fussiness by using these strategies, rather than food to soothe, may calm infants while preventing unnecessary feedings in the absence of hunger and thus overfeeding.

The measurement of food to soothe has typically relied on retrospective reports of maternal use of food to soothe in different general contexts [5]. Recall bias is one limitation of self-report measures that can compromise the accuracy of these data [16]. Further, new parents face unique challenges (e.g. sleep deprivation) that could exacerbate recall bias and contribute to greater inaccuracy [17]. Ecological momentary assessment (EMA) involves repeated sampling of behaviors in real time, in natural environments, and can characterize patterns of behavior change across time and specific context [18]. Using EMA to measure food to soothe in settings where infants eat, sleep, and cry has advantages such as minimizing recall bias, maximizing ecological validity, and capturing dynamic behaviors as they unfold [18].

The **I**ntervention **N**urses **S**tart **I**nfants **G**rowing on **H**ealthy **T**rajectories (INSIGHT) study is a randomized, clinical trial designed to prevent rapid infant weight gain and overweight during early childhood [19]. INSIGHT is based on a responsive parenting framework and emphasized behaviors to encourage responsive feeding, such as using alternatives to food to soothe as a first response to infant distress. Previous reports from INSIGHT have shown that responsive parenting group infants had reduced rapid weight gain during infancy and a lower body mass index at age 1 and 3 years compared with controls [20, 21]. INSIGHT also improved infants’ dietary patterns [22] and feeding practices [23]. For the current analysis, we describe a subset of INSIGHT participants that participated in EMA to capture detailed patterns of soothing strategies mothers used when their infants were fussy. Our first aim was to describe the frequency of different soothing strategies used. Next, we tested the effects of the responsive parenting intervention on maternal use of food to soothe during early infancy. We hypothesized that control mothers would report using food to soothe more often than responsive parenting mothers. Last, we explored possible moderators on whether maternal use of food to soothe differed by time of day, the frequency of infant fussiness, and/or feeding mode. We hypothesized a greater use of food to soothe during the nighttime, when infants fussed less frequently, and for infants not predominantly breastfed.

## Methods

### Participants

Mothers were recruited by research staff from the maternity ward of one hospital (Penn State Milton S. Hershey Medical Center, Hershey, Pennsylvania) shortly after giving birth. Enrollment occurred from January 2012 to March 2014. The eligibility criteria included singleton infants that were full-term (≥37 weeks gestation) and ≥ 2500 g at delivery. Mothers had to be primiparous, English-speaking, and ≥ 20 years of age. Two weeks after delivery, enrolled mothers were randomized into a responsive parenting or control group, stratified by intended feeding mode (breast or formula) and birth weight for gestational age (<50th or ≥ 50th). Additional details on the INSIGHT protocol are published elsewhere [19]. A subsample of INSIGHT participants completed the EMA data collection used in these analyses (*n* = 157), which is 56.3% of the 279 total participants that were randomized and received the first nurse home visit. Because a number of participants found the EMA portion of the study burdensome, the collection of EMA data was discontinued in the interest of long-term retention of participants. This study was approved by the Human Subjects Protection Office at Penn State College of Medicine prior to enrollment of the first participant.

### Intervention and study design

The INSIGHT responsive parenting curriculum taught parents to respond promptly, contingently, and in developmentally appropriate ways to infant cues across 4 behavioral states: drowsy, sleepy, fussy, and alert/calm [19]. The control group curriculum taught home safety guidance to parents (e.g. fire safety, back to sleep), focused around the same 4 infant behavioral states. The responsive parenting and control curriculum were matched for content intensity. At 2 weeks of age, all participants were mailed a packet with information on infant feeding do’s and don’ts. Following this, trained research nurses delivered intervention materials in each family’s home when infants were age 3 weeks.

#### Feeding-focused intervention curriculum

One focus of the INSIGHT curriculum was to promote infant self-regulation of food intake by using responsive feeding techniques. A detailed description of the feeding-specific messages can be found elsewhere [23]. Briefly, parents in the responsive parenting group were taught to recognize hunger and fullness cues, to not pressure infants to eat (e.g. not to finish the bottle), and to not feed as the first response to infant fussiness. When infants were fussy, yet not showing signs of hunger, parents in the responsive parenting group were instructed to first try soothing strategies such as the “5 S’s” – swaddle, shush, swing, side/stomach position, and suck (e.g. give pacifier) [24]. To illustrate these soothing techniques, parents watched the Happiest Baby on the Block DVD (THB Media, LLC) [24] and practiced these techniques with their research nurse during the 3-week home visit. Parents were instructed to first calm their baby using a combination of these techniques, and if their child was showing signs of hunger after being calmed, then to feed. Therefore, parents did not restrict a required feeding, but only fed when infants showed signs of hunger to prevent overfeeding. During the nighttime, parents were instructed to let infants calm on their own. If this did not work, they were instructed to first try “low-level” soothing strategies that did not involve picking the infant up (e.g. rub or pat) before trying “high-level” soothing strategies that did involve picking the infant up (e.g. rocking). This guidance was given to provide more opportunities for infants to develop self-soothing abilities, rather than rely on parent involvement when awake in the night.

### Measures

Demographic information, such as race/ethnicity, income, and marital status were collected at enrollment. Data on maternal age, infant sex, birth weight and length, and gestational age at birth were extracted from medical records.

#### EMA survey

**Fig. 1:**
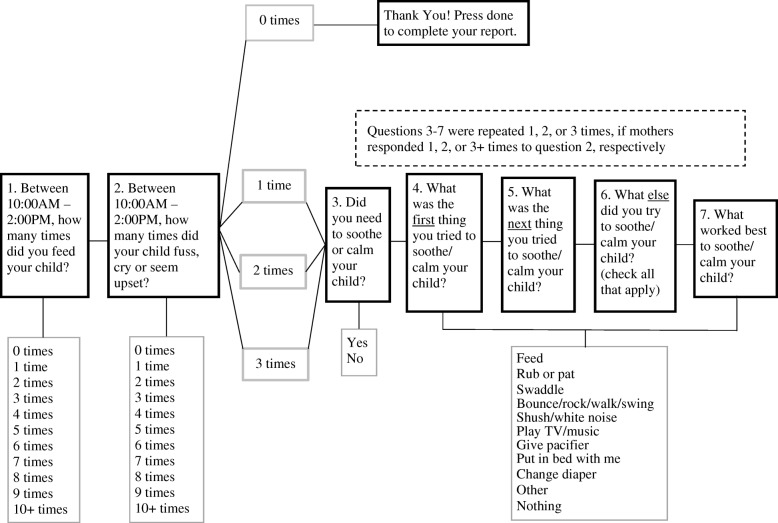
Example of a series of questions asked in the EMA survey, developed for the INSIGHT study, and delivered using smartphones. The clock times in questions 1 and 2 changed to reflect each 4-h block of time within the day

Mothers completed the EMA survey when infants were ages 3 and 8 weeks. These timepoints were chosen, given that 3 weeks was close to the start of the intervention and immediately followed the first nurse home visit. The 8-week timepoint was multiple weeks later and at an age when infant crying peaks. In addition, at 8 weeks, the majority of mothers had not yet returned to work full-time and were able to answer surveys on their infants’ behaviors throughout the day. Mothers were given a study-specific smartphone (LG Fathom VS750) programmed with software (Mobile Survey Development Toolkit) customized for this study. The smartphones beeped 5 times per day, every day, for 5–8 days, until 25 assessments were completed. Participants answered each beep by responding to a series of questions shown in Fig. 1. The 5 beeps occurred at 8:00 AM, 10:00 AM, 2:00 PM, 6:00 PM, and 10:00 PM each day. The survey questions were tailored for each beep to ask about the previous 4 h. For example, the 10:00 AM beep asked about the hours of 6:00 AM – 10:00 AM on that same day. The only exception was the 8:00 AM beep, which asked about the hours of 10:00 PM – 2:00 AM and 2:00 AM – 6:00 AM, so that participants did not have to respond to beeps in the middle of the night.

**Fig. 2:**
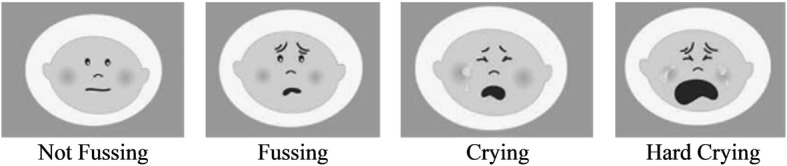
Image provided to mothers to help them identify infants’ fussy events. A fussy event was considered fussing, crying, or hard crying, corresponding to the faces above

The participants were asked to carry the study smartphones with them throughout the day, in order to answer the questions immediately after each beep. If the questions were not answered within 1 h following a given beep, it was considered a missed response. At each beep, participants provided information on soothing strategies used for a maximum of 3 fussy bouts that occurred in the previous 4 h. If an infant fussed more than 3 times in the prior 4 h, soothing strategy data were only collected on the first 3 fussy bouts (see Fig. 1). A fussy bout was considered infant fussing, crying, or hard crying, as illustrated in Fig. 2. Prior to starting the EMA survey, this figure was provided to mothers in an instructional hand-out in order to standardize what mothers considered to be a fussy bout. Service to the phone was suspended once participants answered 25 beeps or 8 days had passed, whichever came first.

#### Categories of soothing strategies

As shown in Fig. 1, mothers reported which soothing strategy they used first, second, and all other strategies used for each fussy bout*.* Food to soothe was subsequently characterized in two ways. First, each fussy bout was labeled as either 1) Fed First or 2) Not Fed First. *Fed First* occurred if mothers reported feeding as the first strategy to soothe/calm a given fussy bout (Fig. 1, Question 4); *Not Fed First* occurred if feeding was not the first strategy used. This included situations where feeding was used, but not as the first strategy, or where feeding was not used at all. This categorization aligned with the responsive parenting curriculum given that even when infants were fussy due to hunger reasons, mothers in the responsive parenting group were instructed to not feed as the first response. Second, among the *Not Fed First* bouts, we further identified these as 1) Fed Later or 2) Not Fed. *Fed Later* occurred if mothers reported feeding as the second or later strategy used to soothe/calm a given fussy bout (Fig. 1, Questions 5 and 6); *Not Fed* occurred if mothers did not report feeding as any strategy used (Fig. 1, Questions 4–6).

If mothers reported rub or pat, swaddle, bounce/rock/walk/swing, shush/white noise, or pacifier as the first soothing strategy used, this was considered a responsive parenting strategy. If mothers reported using one of the other soothing strategies first (feed, play TV/music, put in bed with me, change diaper, other, or nothing), this was considered as *not* using a responsive parenting strategy. During the nighttime only, if mothers reported using swaddle or bounce/rock/walk/swing as the first soothing strategy used, this was considered a “high-level” soothing strategy (e.g. involved picking the infant up). If mothers reported using rub or pat, shush/white noise, pacifier, or TV/music, this was considered a “low-level” soothing strategy (e.g. did not involve picking the infant up). Last, we considered changing the infant’s diaper as a biological need, rather than a soothing strategy; therefore, “change diaper” was removed post hoc from the response options of first soothing strategies. If mothers reported “change diaper” as the first soothing strategy used, then the second reported soothing strategy was considered to be the first soothing strategy used.

#### Infant fussiness

Infant fussiness was quantified as the number of infant fusses reported during each 4-h interval. At each cell phone beep, mothers reported how many times their infant fussed in the previous last 4 h (Fig. 1, Question 2), which was treated as a continuous variable (0–10), corresponding to the response options of 0–10+, respectively.

#### Feeding mode

Infant feeding mode was collected at 2 weeks of age using the Infant Food Frequency Questionnaire, which was modified from the Harvard Service Food Frequency Questionnaire [25]. Mothers reported on the number of feeds per day that were breast milk and/or formula. Infants were considered predominantly breastfed if ≥80% of feedings were breast milk, either at the breast or bottle. If < 80% of feedings were breast milk, infants were considered not predominantly breastfed.

### Data analysis

We used a series of generalized linear models (GLMs) in the generalized estimating equations framework to examine factors related to the number of infant fusses and feeds at a descriptive level. A repeated measures GLM was fitted to compare the numbers of responses across these factors.

Two repeated-measures GLMs (one for feeding and one for fussing) grouped by mother-infant dyad examined the relationship between infant age and the number of times infants fussed or were fed over a four-hour interval, without including time of day or study group. These models accounted for repeated measures within the same mother-infant dyad to account for within-dyad dependencies. A second pair of models examined the relationship between time of day (e.g. 6:00 PM – 10:00 PM) and fussing/feeding behavior to examine diurnal patterns, without including study group or infant age. A third pair of models examined the relationship between study group and fussing/feeding behavior, without regard to time of day or infant age. Next, a series of GLMs were used to test the relationships of these same predictors (infant age, time of day, and study group) on the proportion of fusses that needed to be soothed and the number of soothing strategies used for each fuss. A Poisson link function was not used for models including count data, as the distribution of these data were relatively normal. To ensure this was appropriate, models were also run using a Poisson link function and no differences in the pattern of results were found.

A second series of repeated-measures GLMs was used to test the influence of infant age, time of day, and study group on the use of food to soothe. For these models, time of day was reduced to two categories: daytime (6:00 AM – 10:00 PM) and nighttime (10:00 PM – 6:00 AM). The duration for nighttime was chosen to most closely resemble when parents were asleep. We fitted these models to answer the question if infants were fed as the first soothing strategy (coded as a dichotomous variable: Fed First/Not Fed First). An initial test for a three-way interaction was not significant, so the three-way interaction was removed from the model. Of the possible two-way interactions, study group by infant age and infant age by time of day were significant. We then tested for the main effects of study group and time of day within each age group.

Next, we examined fussy bouts in which mothers did *not* feed first. These bouts were further divided into those in which infants were Fed Later vs. Not Fed in response to this fussy bout. Instances where mothers fed first were not included. This model followed a similar procedure as the preceding model: tests of the higher-level interactions were performed first and removed if non-significant. In this case, only the infant age by time of day interaction was retained.

To examine the relationship of infant fussiness on the use of food to soothe, we repeated the previous two models while including the number of fussy bouts during each time period and feeding mode (predominantly breastfed or not predominantly breastfed) as a predictor. Study group, infant age, and time of day were tested as possible moderators using interaction terms. Again, higher-level interactions were tested first and removed if not significant. Infant age and time of day, but not study group, were significant moderators.

GLMs used *proc genmod* in SAS Version 9.4 (SAS Institute Inc., Cary, NC), with statistical significance defined a priori with a cutoff of *p* < .05. For binary outcomes we used a logistic link function. Results are reported as mean ± standard deviation or odds ratios (OR) with 95% confidence intervals (CI) for logistic analyses.

## Results

Participants were predominantly White, married, and college educated (Table 1). About 73% of participants reported an annual household income ≥$50,000 (Table 1). For the EMA survey, participants responded to an average of 24.5 ± 1.8 total beeps at 3 weeks and 24.2 ± 2.4 total beeps at 8 weeks, indicating high compliance. There was no difference in compliance by study group or infant age (*p* > 0.05).

**Table 1:** Participant demographics by study group (*n* = 157)

	Responsive Parenting (*n* = 81)	Control (*n* = 76)
**Maternal demographics**		
Age (years), mean (SD)	28.6 (4.4)	28.6 (4.9)
Pre-pregnancy BMI (kg/m^2^), mean (SD)	25.3 (4.7)	24.8 (4.6)
Gestational weight gain (kg), mean (SD)	15.5 (6.6)	16.1 (6.8)
Hispanic/Latino, n (%)	5 (6.3)	5 (6.6)
Race, n (%)		
Black	7 (8.6)	4 (5.3)
White	68 (84.0)	72 (94.7)
Native Hawaiian/Pacific Islander	1 (1.2)	0 (0)
Asian	3 (3.7)	0 (0)
Other (Multi-Racial)	2 (2.5)	0 (0)
Education, n (%)		
High school or less	7 (8.6)	9 (11.8)
Some college	21 (25.9)	26 (34.2)
College graduate	30 (37.0)	27 (35.5)
Graduate degree +	23 (28.4)	14 (18.4)
Married, n (%)	57 (70.4)	56 (73.7)
Annual household income, n (%)		
< $10,000	2 (2.5)	3 (4.0)
$10,000-24,999	6 (7.4)	5 (6.6)
$25,000-49,999	2 (2.5)	13 (17.1)
$50,000-74,999	29 (35.8)	17 (22.4)
$75,000-99,999	21 (25.9)	16 (21.1)
≥ $100,000	14 (17.3)	17 (22.4)
Do not know/refused to answer	7 (8.6)	5 (6.6)
**Infant demographics**		
Male sex, n (%)	41 (50.6)	34 (44.7)
Gestational age (weeks), mean (SD)	39.7 (1.2)	39.5 (1.2)
Birth weight (kg), mean (SD)	3.4 (0.4)	3.5 (0.4)
Birth length (cm), mean (SD)	51.0 (2.3)	51.1 (2.0)

### Description of infant fusses and feeds

Mothers reported that infants fussed 2.4 ± 1.3 times/4-h interval at 3 weeks and 2.2 ± 1.3 times/4-h interval at 8 weeks (*p* < 0.01). Infants were fed 2.0 ± 0.8 times/4-h interval at 3 weeks and 1.8 ± 0.8 times/4-h interval at 8 weeks (*p* < 0.01). The number of fusses and feeds within a 4-h interval differed by time of day (*p* < 0.01). Averaged across the two infant ages, the most fusses and feeds occurred between 6:00 PM – 10:00 PM (2.6 ± 1.5 fusses; 2.1 ± 0.9 feeds), while the least occurred between 2:00 AM – 6:00 AM (1.8 ± 1.0 fusses; 1.6 ± 0.8 feeds), compared to all other time points within a day (*p* < 0.05). The number of fusses and feeds within a given 4-h interval did not differ by study group (*p* > 0.05).

### Soothing strategies used

Mothers reported that 87.3 ± 14.6% of all fusses needed to be soothed, which did not differ by study group or infant age (*p* > 0.05 for both). Among fusses that needed to be soothed, mothers reported using an average of 2.5 ± 1.0 (range: 0–6.5) different soothing strategies, which did not differ by study group (*p* > 0.05). Mothers reported using more soothing strategies between 6:00 PM – 10:00 PM (2.7 ± 1.0) and 10:00 PM – 2:00 AM (2.6 ± 1.2), than between 2:00 AM – 6:00 AM (2.4 ± 1.2) (*p* < 0.05).

The distribution for the proportion of soothing strategies used first differed by study group (*p* < 0.01). Overall, feeding was the most common strategy used first. Half (50.7%) of all fusses from control infants and 37.8% from responsive parenting infants were soothed by feeding first. The next most common strategy used first was bounce/rock/walk/swing (Control: 24.1% of fusses, Responsive parenting: 23.1% of fusses) while play TV/music and put in bed with me were rarely used (0.4–0.6% of fusses).

### Intervention differences on soothing strategies

**Fig. 3:**
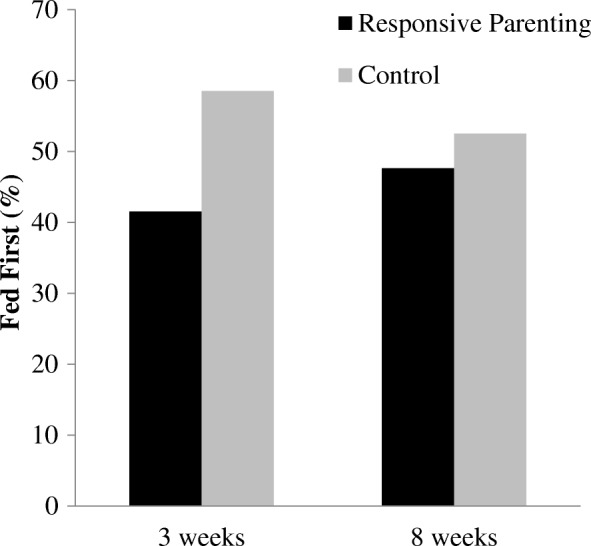
An interaction of study group by infant age on feeding first indicated the control group had greater odds than the responsive parenting group of feeding first at 3 weeks (*p* < 0.01), and this effect decreased over time at 8 weeks (*p* = 0.053)

There was a significant study group by infant age interaction on the outcome of Fed First vs. Not Fed First (*p* = 0.02; Fig. 3). The control group had greater odds of having Fed First than the responsive parenting group at 3 weeks, with a strong trend at 8 weeks (3 weeks: OR = 1.9; 95% CI = 1.4–2.7; *p* < 0.01; 8 weeks: OR = 1.4; 95% CI = 1.0–2.1; *p* = 0.053). More responsive parenting mothers reported using a responsive parenting intervention strategy first, before feeding, than control mothers at 3 weeks (58.1% vs. 41.9%, respectively; *p* < 0.01) and 8 weeks (57.1% vs. 42.9%, respectively; *p* < 0.01). Similarly, during the nighttime (10:00 PM – 6:00 AM), significantly more responsive parenting mothers reported using a “low-level” soothing strategy before feeding or a “high-level” soothing strategy at 3 weeks (60.0% vs. 40.0%, respectively; *p* < 0.01), but not 8 weeks (52.6% vs. 47.4%, respectively; *p* = 0.20).

Feeding was the soothing strategy that mothers reported worked best. When examining this by study group, fewer fusses from responsive parenting group infants (49.5%), then controls (61.0%), were soothed best by feeding (*p* < 0.01). The next best soothing strategy was bounce/rock/walk/swing (Responsive parenting: 15.6%, Control: 14.7% of fusses; difference *p* = 0.45). Soothing strategies that mothers reported rarely worked best included play TV/music (Responsive parenting: 0.3%, Control: 0.7% of fusses; *p* = 0.02) and put in bed with me (Responsive parenting: 1.5%, Control: 1.3% of fusses; *p* = 0.48).

### Indicators of using food to soothe

#### Time of day

There was a significant time of day by infant age interaction on Fed First vs. Not Fed First (*p* = 0.03). At ages 3 and 8 weeks, the odds of having Fed First were greater during the nighttime than during the daytime, with this effect increasing over time (Table 2). There was no study group by time of day interaction (*p* = 0.28).

**Table 2:** Greater odds for mothers having fed as the first soothing strategy in response to infant fussiness during the nighttime, compared to the daytime. Models show time of day by study group and time of day by infant age interactions on Fed First, compared to Not Fed First

	Time of day by infant age interaction			
	OR (95% CI)	*p* value	OR (95% CI)	*p* value
	3 weeks (*n* = 153)		8 weeks (*n* = 144)	
Daytime	REF		REF	
Nighttime	1.6 (1.4–1.8)	< 0.01	2.1 (1.7–2.6)	< 0.01

*REF* Reference, *OR* Odds Ratio, *CI* Confidence Interval

Daytime = 6:00 AM-10:00 PM; Nighttime = 10:00 PM-6:00 AM

**Fig. 4:**
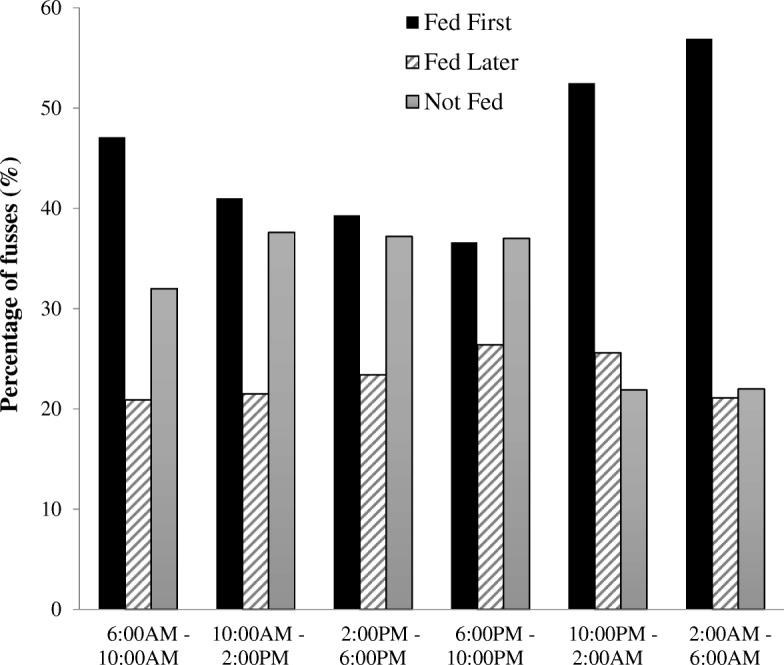
A greater percentage of fussy bouts between 10:00 PM – 2:00 AM and 2:00 AM – 6:00 AM resulted feeding as the first response to infant fussiness (Fed First), rather than feeding not as the first response (Fed Later) or not feeding as any response (Not Fed)

Next, among the subset of fussy bouts where mothers did *not* feed first, we looked at indicators of Fed Later vs. Not Fed. Parents had greater odds of having Fed Later during the nighttime, compared to the daytime, at both 3 and 8 weeks (3 weeks: OR = 1.4, 95% CI = 1.2–1.7; *p* < 0.01; 8 weeks: OR = 2.2; 95% CI = 1.7–2.8; *p* < 0.01). There were no study group differences in Fed Later, compared to Not Fed (*p* = 0.48). Figure 4 shows the percentage of fussy bouts that resulted in Fed First, Fed Later, and Not Fed First for each 4-h interval within a given day.

#### Frequency of infant fussiness

The association between infant fussiness and being Fed First differed by infant age (*p* = 0.01). Parents had greater odds of having Fed First during periods when infants fussed less, with this effect increasing over time (Table 3). The relationship of infant fussiness to Fed First, compared to Not Fed First, also differed by time of day (*p* = 0.04). Parents had greater odds of having Fed First during periods when infants fussed less, with this effect being greater in the nighttime than in the daytime (Table 3). There were no interactions on the relationship between infant fussiness and Fed Later, compared to Not Fed (*p* > 0.05); however, a main effect of infant fussiness revealed that overall, parents had greater odds of having Fed Later (OR = 1.2; 95% CI = 1.1–1.3; *p* < 0.01), compared to Not Fed, during periods when infants fussed less.

**Table 3:** Greater odds for mothers having fed as the first soothing strategy in response to infant fussiness during periods of the day when infants fussed less frequently. Models show infant fussiness by infant age and infant fussiness by time of day interactions on Fed First, compared to Not Fed First

Infant fussiness by infant age interaction				
	OR (95% CI)	*p* value	OR (95% CI)	*p* value
	3 weeks (*n* = 153)		8 weeks (*n* = 144)	
^a^# infant fusses	1.2 (1.1–1.3)	< 0.01	1.3 (1.2–1.4)	< 0.01
Infant fussiness by time of day interaction				
	OR (95% CI)	*p* value	OR (95% CI)	*p* value
	Daytime (*n* = 157)		Nighttime (*n* = 156)	
^a^# infant fusses	1.2 (1.1–1.3)	< 0.01	1.3 (1.2–1.5)	< 0.01

*OR* Odds Ratio, *CI* Confidence Interval

^a^frequency of infant fusses within a given 4-h period of the day. Daytime = 6:00 AM-10:00 PM; Nighttime = 10:00 PM-6:00 AM

#### Feeding mode

At 2 weeks of age, 60.9% of infants were predominantly breastfed while 39.1% were not, which did not differ by study group (*p* = 0.38). Feeding mode moderated the intervention effect on Fed First vs. Not Fed First (*p* = 0.04). Among predominantly breastfed infants, the control group had greater odds of having Fed First, compared to the responsive parenting group (Table 4). The use of Fed First did not differ by treatment group among infants who were not predominantly breastfed. Similarly, feeding mode moderated the relationship of time of day on Fed First vs. Not Fed First (*p* = 0.03). For predominantly breastfed infants, there were greater odds of having Fed First during the nighttime, compared to the daytime, while for infants not predominantly breastfed, the use of Fed First did not differ by time of day (Table 4). Feeding mode did not moderate the relationship of infant age on Fed First vs. Not Fed First (*p* = 0.33) or the relationship of study group, time of day, or infant age on Fed Later vs. Not Fed (*p* > 0.05).

**Table 4:** Among predominantly breastfed infants, there were greater odds for feeding as the first soothing strategy in response to infant fussiness for Control mothers, compared to Responsive Parenting mothers, and during the nighttime compared to the daytime. Models show study group by feeding mode and time of day by feeding mode interactions on Fed First, compared to Not Fed First

	OR (95% CI)	*p* value	OR (95% CI)	*p* value
	Predominantly breastfed (*n* = 95)		Not predominantly breastfed (*n* = 61)	
Study group by feeding mode interaction				
Responsive Parenting	REF		REF	
Control	1.2 (1.1–1.3)	< 0.01	1.0 (0.9–1.2)	0.70
Time of day by feeding mode interaction				
Daytime	REF		REF	
Nighttime	1.1 (1.0–1.1)	< 0.01	1.0 (1.0–1.0)	0.69

Predominantly breastfed: ≥80% feedings as breast milk, either at the bottle or breast. Nighttime = 10:00 PM-6:00 AM; Daytime = 6:00 AM-10:00 PM

*OR* Odds Ratio, *CI* Confidence Interval

## Discussion

INSIGHT’s responsive parenting intervention reduced mothers’ use of food to soothe and increased their use of alternative soothing strategies as the first response to infant fussiness. Mothers in the responsive parenting group had lower odds of feeding first at infant ages 3 and 8 weeks and reported fewer fusses were soothed best by feeding compared with the control group. In accordance with the INSIGHT responsive parenting curriculum, more responsive parenting mothers used a responsive parenting intervention strategy first, before feeding, at both 3 and 8 weeks. During the night at 3 weeks, more responsive parenting mothers also used a “low-level” soothing strategy first, before a “high-level” soothing strategy or feeding.

This study is the first to use EMA to capture detailed patterns of food to soothe in real time and demonstrate positive outcomes of a responsive parenting intervention on reducing maternal use of food to soothe during an infant’s first two months of life. Our results show the responsive parenting group had lower odds of feeding first in response to infant fussiness at 3 weeks, and at 8 weeks these intervention effects showed a similar trend. Previously, we reported positive intervention results on reducing food to soothe at ages 16 and 28 weeks, when measured using the Baby’s Basic Needs Questionnaire [23]. During the nurse home visits at infant ages 3, 16, and 28 weeks, responsive parenting mothers were taught to use alternative soothing strategies before feeding. Parents were encouraged to calm their infant first, look for signs of hunger, and then feed if their infant demonstrated hunger cues [19]. Feeding as a first response to infant fussiness was successfully reduced at ages in which this responsive feeding guidance was delivered. Given the observational data suggesting food to soothe is associated with greater weight status [5, 6, 9], our results provide evidence that reducing food to soothe may partially explain our findings that responsive parenting group infants had less rapid weight gain between birth and 6 months [20].

When examined by feeding mode, the intervention effects of responsive parenting mothers having lower odds of having fed first compared to control mothers persisted for infants who were predominantly breastfed, but not for infants who were not predominantly breastfed at 2 weeks of age. The INSIGHT responsive parenting curriculum may have been particularly salient for predominantly breastfeeding mothers, while additional or modified intervention messaging may be needed for mothers not predominantly breastfeeding. It should be noted that feeding mode was quantified as what infants were fed (breast milk vs. formula), rather than how infants were fed (at the breast vs. bottle), and future studies should look at how infants are fed on mothers use of food to soothe. Further, feeding mode for these analyses was quantified when infants were 2 weeks of age. It is possible that predominantly breastfed infants at 2 weeks of age were no longer predominantly breastfed at 8 weeks of age when the EMA survey on using food to soothe was completed.

We also showed (in agreement with earlier findings from self-reported data [23]) that fewer responsive parenting group mothers than controls reported feeding as having worked best at soothing their infants. The more frequent use of food to soothe among control group infants may have developed into a learned behavior that contributed to its effectiveness. Prior research among preschool aged children found that eating in response to negative emotions was a learned behavior that was largely influenced by the environment [26]. Decreasing the use of food to soothe might prevent this learned association of eating when distressed. More responsive parenting mothers used alternative soothing strategies first, rather than feeding, and were less likely to report feeding as the strategy that worked best. Therefore, INSIGHT’s responsive parenting guidance delivered during early infancy not only reduced the use of food to soothe but also its perceived effectiveness.

At the 3-week assessment point, more responsive parenting mothers used a “low-level” soothing strategy (e.g. did not involve picking the infant up), before a “high-level” strategy or feeding, during the night. “High-level” soothing strategies are thought to be non-adaptive since infants cannot recreate these strategies on their own [27]. The repetitive use of these non-adaptive strategies may reduce self-soothing ability and result in more frequent night wakings that require parent involvement [28, 29]. Our study is the first to test these alternative soothing strategies in the context of a responsive parenting intervention. While more empirical research is needed on these soothing strategies, we theorize the routine use of the responsive parenting intervention or “low-level” soothing strategies first, rather than “high-level” or feeding, contributes to the development of greater infant self-regulation. By using these alternative soothing strategies first, infants learn that food is not the first, most immediate response to distress. This may translate into the greater use of non-food related strategies to self-soothe negative emotions in later life. What also remains unknown are the effects of routinely feeding to soothe, but not as a first strategy (Fed Later) vs. not feeding at all (Not Fed) in response to fussiness. Both teach infants to use non-food related strategies to self-soothe when distressed; yet, feeding later may have fewer protective benefits on the development of obesity. Future research should continue to explore how using alternative soothing strategies before feeding differs from feeding first or not at all, in response to infant fussiness.

A greater use of food to soothe has been shown among mothers who perceive their infants as having a more negative or fussy temperament [5–8]. To build upon this, we compared maternal use of food to soothe during times of the day in which infants fussed more frequently. This allowed for a within-infant comparison of fussy and less fussy times, rather than a between-infant comparison based on temperament. Mothers were more likely to feed first during times of the day when infants fussed less frequently. Mothers reported the greatest number of fusses occurred between 6:00 PM– 10:00 PM with 36.6% resulting in Fed First, while the least number of fusses occurred between 2:00 AM – 6:00 AM with 56.9% resulting in Fed First. When infants fuss, after not fussing for the previous few hours, mothers may be more likely to perceive that fuss as a signal that the infant is hungry. Mothers may have realized it was time for their infant to eat after the longer interval without fussing. In contrast, when infants fuss, after having fussed repeatedly in the previous few hours, mothers may be more likely to think their fussiness is due to reasons other than just hunger. In this case, mothers were more likely to try different soothing strategies for each of the frequent fusses. These findings indicate that mothers may recognize the last time infants fussed and use this to discriminate when to feed or not feed as the first soothing strategy. Further, there were greater odds of feeding first in the nighttime, as compared to the daytime. At this young of an age, infants should be fed every few hours; therefore, overnight feedings are necessary. However, even so, parents are encouraged to first calm their infant, before feeding. If the use of feeding first in response to nighttime fussiness were to persist as infant’s age when overnight feedings become less necessary, this could result in excess feedings and more frequent sleep disruptions [30].

One of the greatest strengths of this analysis was the EMA study design; yet, this method has limitations. The intensive nature of collecting survey data every 4 h, across multiple days, increased participant burden. At the time of data collection, a separate mobile device with EMA software was given to mothers to be carried with them throughout the day. Mothers reported that carrying this extra device with them and the frequent ringing was demanding. Consequently, the EMA data was collected in just over half of the INSIGHT sample. With the recent developments in technology, EMA software is now available for personal mobile devices and platforms such as REDCap [31, 32]. We hypothesize using EMA software on these platforms, rather than a separate mobile device, will ease the burden that our participants experienced and encourage greater participation in future studies. Another limitation of these data was that information on when infants’ displayed hunger cues was not obtained. Future work should collect information on infants’ hunger cues to disentangle feeding in response to fussy bouts when infants were hungry versus when they were not hungry. Further, the repetitive nature of EMA greatly reduced recall bias; yet, this self-reported measure still allows for the possibility of response bias. Our results should be interpreted in the context that mothers may have answered the EMA survey in a way that they felt was more compliant with the guidance they received. Last, the study population was predominantly White, English speaking, first-time mothers who were well educated. Findings cannot be generalized to other populations.

## Conclusion

Overall, the INSIGHT responsive parenting intervention was successful at reducing maternal use of feeding as the first response to infant distress early in life. The responsive parenting intervention also reduced the frequency in which feeding was considered the most effective strategy at soothing infant fussiness. Educating parents on responsive parenting behaviors at the start of infancy can effectively modify parenting practices around infant fussing and feeding. Clinical guidance and intervention messaging should consider these findings and continue to encourage mothers to try alternative soothing strategies before feeding when infants show signs of hunger. Early infancy is an opportune time to establish this behavior given the frequency at which fussing and feeding naturally occur. The use of alternative soothing strategies, rather than feeding first, has the potential to improve infants’ self-regulation and weight gain trajectory. To continue this work, future analyses should test if reducing food to soothe during infancy improves weight status and obesogenic eating behaviors, such as emotional eating, in later childhood.

## Data Availability

The datasets used and/or analyzed during the current study are available from the corresponding author on reasonable request.

## Abbreviations

CI: Confidence interval
EMA: Ecological momentary assessment
GLMs: Generalized linear models
INSIGHT: Intervention Nurses Start Infants Growing on Healthy Trajectories
OR: Odds ratios
